# Evaluation of a Potential Bacteriophage Cocktail for the Control of Shiga-Toxin Producing *Escherichia coli* in Food

**DOI:** 10.3389/fmicb.2020.01801

**Published:** 2020-07-24

**Authors:** Nicola Mangieri, Claudia Picozzi, Riccardo Cocuzzi, Roberto Foschino

**Affiliations:** Department of Food, Environmental and Nutritional Sciences, Università degli Studi di Milano, Milan, Italy

**Keywords:** bacteriophage, Shiga-toxin producing *Escherichia coli*, biocontrol, antibiotic resistance, food safety

## Abstract

Shiga-toxin producing *Escherichia coli* (STEC) are important foodborne pathogens involved in gastrointestinal diseases. Furthermore, the recurrent use of antibiotics to treat different bacterial infections in animals has increased the spread of antibiotic-resistant bacteria, including *E. coli*, in foods of animal origin. The use of bacteriophages for the control of these microorganisms is therefore regarded as a valid alternative, especially considering the numerous advantages (high specificity, self-replicating, self-limiting, harmless to humans, animals, and plants). This study aimed to isolate bacteriophages active on STEC strains and to set up a suspension of viral particles that can be potentially used to control STEC food contamination. Thirty-one STEC of different serogroups (O26; O157; O111; O113; O145; O23, O76, O86, O91, O103, O104, O121, O128, and O139) were investigated for their antibiotic resistance profile and sensitivity to phage attack. Ten percent of strains exhibited a high multi-resistance profile, whereas ampicillin was the most effective antibiotic by inhibiting 65% of tested bacteria. On the other side, a total of 20 phages were isolated from feces, sewage, and bedding material of cattle. The viral particles proved not to carry genes codifying Shiga-toxins and intimin. No STEC was resistant to all phages, although some strains revealed weak sensitivity by forming turbid plaques. Three different bacteriophages (forming the “cocktail”) were selected considering their different RAPD (Random Amplification of Polymorphic DNA) profiles and the absence of virulence-encoding genes and antibiotic-resistance genes. The lytic ability against STEC strains was investigated at different multiplicity of infection (MOI = 0.1, 1, and 10). Significant differences (*p* < 0.05) among mean values of optical density were observed by comparing results of experiments at different MOI and controls. An effective reduction of bacterial population was obtained in 81% of cases, with top performance when the highest MOI was applied. The efficacy of the phage cocktail was tested on fresh cucumbers. Results showed a reduction in pathogenic *E. coli* by 1.97–2.01 log CFU/g at 25°C and by 1.16–2.01 log CFU/g at 4°C during 24 h, suggesting that the formulated cocktail could have the potential to be used in bio controlling STEC different serogroups.

## Introduction

Certain strains of *Escherichia coli*, a bacterium that is naturally resident in the human gut, can cause gastrointestinal diseases, bloody diarrhea that can develop in complex illnesses as hemorrhagic colitis (HC) and hemolytic-uremic syndrome (HUS) (Kaper et al., 2004). These strains, characterized by the production of Shiga toxins and often abbreviated as STEC (Shiga toxin-producing *E. coli*), have ruminants as major reservoir. The most common route of transmission to humans is *via* undercooked contaminated meats or fresh dairy products from raw milk (Karmali et al., 2010). The last European Union One Health 2018 Zoonoses Report (EFSA and ECDC, 2019) indicated STEC infections in humans as the third most commonly reported zoonosis in the EU with a notification rate increased by 39.0% compared with 2017. Serotype O157:H7 is still the most common one related to human illness, but non-O157 strains, and in particular O26, O103, and O91, are increasing in importance (Croxen et al., 2013; EFSA and ECDC, 2019).

With these data in mind, it is easy to understand the importance to improve techniques for the control of STEC for food safety and consumer protection. New approaches such as radiation, high pressure, pulsed electric field, and ultrasound are quite expensive and sometimes can not be applied to fresh and ready-to eat products. Instead, the use of bacteriophage for reducing microbial pathogens in food is well established (Moye et al., 2018) and in 2009 the European Food Safety Authority (EFSA) reported that bacteriophages can be very effective in the elimination of pathogens from meat, milk, and dairy products (EFSA, 2009).

The benefit of using bacteriophages as biocontrol instruments far outweigh the drawbacks. In fact, phages are highly active and specific; harmless to humans, animals and plants; mostly able to resist to food processing stressors; self-replicating and self-limiting. Furthermore, bacteriophages are abundant in food indicating that phages can be found in the same environment of their bacterial host and daily ingested by humans and animals providing evidence of no harmful effects (McCallin et al., 2013). Finally, they do not affect texture, taste, smell, and color of food and they have proved to extend shelf life and in some cases, they showed to lyse the host cells even at temperatures as low as 1°C (Greer, 1988).

According to literature, the principal efforts of using bacteriophages against STEC strains have been directed mainly toward serogroup O157 (O’Flynn et al., 2004; Abuladze et al., 2008; Sharma et al., 2009; Viazis et al., 2011; Hudson et al., 2015; Snyder et al., 2016). However, given the increase in the finding of non-O157 in food, different authors have focused their efforts also toward other serogroups (Tomat et al., 2013; Tolen et al., 2018; Liao et al., 2019).

The aim of this research study is to obtain a phage suspension that can be used against the major number of STEC strains as possible (O157 and non-O157). Ideally, this preparation could be implemented to different stages of production, from disinfection of equipment and contact surfaces (biosanitation) to treatment of raw products and RTE foods (biocontrol).

## Materials and Methods

### Bacterial Strains and Growth Conditions

*Escherichia coli* strains provided from different institutes (ATCC; Istituto di Ispezione degli Alimenti di Origine Animale, Milan, Italy; Collaborative Centre for Reference and Research on *Escherichia* (WHO); Statens Serum Institut (SSI), Denmark; Istituto Superiore di Sanità, Rome, Italy) or collected in previous studies (Picozzi et al., 2016) were used in this work (Table 1). Strains were isolated from human stool, raw goat milk and milking filters. Bacterial cultures were grown aerobically in Luria-Bertani (LB) broth medium (5 g L^–1^NaCl, 5 g L^–1^yeast extract, 10 g L^–1^Tryptone) at 37°C. LB agar plates were prepared with LB broth supplemented with 1.5 or 0.5% Agar (EMD Chemicals, San Diego, CA, United States) for plating bacteria or phage plaque testing, respectively.

**TABLE 1:** STEC strain used in this work with information relative to antibiotic resistance according to EUCAST (2019).

Strain	Serogroup	Antibiotic resistance	Antibiotic sensitivity	MAR index
ATCC35150^a^	O157	AMC, CHL, AMP	CIP, NOR, NAL	0.50
393^b^	O26	CIP, NOR, CHL, NAL	AMC, AMP	0.67
15R^b^	O76	AMC, NAL	CIP, NOR, CHL, AMP	0.34
214CH^c^	O157	CIP, AMP, NAL	AMC, NOR, CHL	0.50
228GS^c^	O145	AMC, CIP, NOR CHL, NAL	AMP	0.83
229RACH^c^	O111	AMC, AMP, NAL	CIP NOR, CHL	0.50
239RA^c^	O26	CHL, NAL	AMC, CIP, NOR, AMP	0.34
243RACH^c^	O26	AMC, CIP, CHL, NAL	NOR, AMP	0.67
243ROI-A^c^	O26	NOR, NAL	AMC, CIP, CHL, AMP	0.34
33C^b^	O23	AMP, NAL	AMC, CIP, NOR, CHL	0.34
380USA^b^	O157	NOR, CHL, NAL	AMC, CIP, AMP	0.50
6182-50^d^	O113	–	AMC, CIP, NOR CHL, AMP, NAL	0
62 19/L^b^	O157	NOR, CHL, AMP, NAL	AMC, CIP	0.67
C679-12^e^	O104	AMC, NOR, CHL	CIP, AMP, NAL	0.50
ED13^f^	O157	CIP, CHL, AMP, NAL	AMC, NOR,	0.67
ED142^f^	O111	CIP, NOR, AMP	AMC, CHL, NAL	0.50
ED161^f^	O86	AMC, CIP, NOR	CHL, AMP, NAL	0.50
ED172^f^	O103	CIP, NOR	AMC, CHL, AMP, NAL	0.34
ED173^f^	O145	CIP, NOR, AMP	AMC, CHL, NAL	0.50
ED226^f^	O113	AMC, CIP, NOR, NAL	CHL, AMP	0.67
ED33^f^	O139	AMC, CIP	NOR, CHL, AMP, NAL	0.34
ED56^f^	O26	CIP, NOR, CHL, NAL	AMC, AMP	0.67
ED76^f^	O91	CIP, NOR, CHL, AMP	AMC, NAL	0.67
ED82^f^	O111	AMC, NOR, AMP, NAL	CIP, CHL	0.67
ED238^f^	O121	CIP, NOR CHL, AMP, NAL	AMC	0.83
F1-1^c^	O26	NAL	AMC, CIP, NOR, CHL, AMP	0.17
F10-4^c^	O26	CIP, NAL	AMC, NOR, CHL, AMP	0.34
F11-4^c^	O26	AMC, CIP, NAL	NOR, CHF, AMP	0.50
F95^c^	O26	AMC, NAL	CIP, NOR, CHL, AMP	0.34
F95-3^c^	O26	–	AMC, CIP, NOR, CHL, AMP, NAL	0.00
PO128c	O128	AMC, CIP, NOR CHL, NAL	AMP	0.83

**^*a*^American Type Culture Collection. ^*b*^Istituto di Ispezione degli Alimenti di Origine Animale (Milan, Italy). ^*c*^Picozzi et al. (2016)*. *^*d*^Collaborative Center for Reference and Research on Escherichia (WHO) (Orskov et al., 1977). ^*e*^Statens Serum Institut (SSI) in Denmark ^*f*^ Istituto Superiore di Sanità (Rome, Italy). AMC, Amoxicillin/clavulanic acid; AMP, Ampicillin; CHL, Chloramphenicol; CIP, Ciprofloxacin; NAL, Nalidixic acid; NOR, Norfloxacin.**

### Antibiotic Resistance Assay

The resistance of STEC strains to antimicrobial compounds was tested by disk diffusion susceptibility test (Matuschek et al., 2014). STEC strains were cultivated in LB broth at 37°C until they reached a concentration of about 5 × 10^8^ cells/mL. Cultures were streaked onto the surface of a LB agar plate, using a sterile cotton swab in three different directions. Sterile paper disks (6 mm in diameter) were applied onto the surface of the plate and spotted with six different antibiotics: ampicillin (AMP 10 μg), chloramphenicol (CHF 30 μg), ciprofloxacin (CIP 5 μg), nalidixic acid (NAL 30 μg), norfloxacin (NOR 10 μg) (Sigma-Aldrich, St. Louis, United States), and amoxicillin-clavulanic acid (AMC 20 μg) (So.Se. Pharm Srl, Pomezia, Italy). For each isolate, the Multiple Antibiotic Resistance (MAR) index, defined as a/b, where a represents the number of antibiotics to which the isolate was resistant, and b represents the number of antibiotics to which the isolate was exposed, was calculated (Krumperman, 1983). Intermediate test results (partial sensitivity) were considered as negative (sensitive). Since chloramphenicol was dissolved in 50% (v/v) ethanol, a disk containing only 50% (v/v) ethanol and no antibiotic was also added as a negative control, together with a disk with sterile water. After incubation overnight at 37°C the diameters of the inhibition zones (mm) were measured and then interpreted as susceptible, intermediate or resistant according to the EUCAST clinical breakpoints (EUCAST, 2019).

### Phage Isolation and Purification

In order to isolate bacteriophages, twenty-two samples were collected from three breeding farms in the area of Milan. Approximately 100 g of feces, bedding material or sewage from cattle and sheep were sampled in sterile 200 mL cup and stored at 4°C until processing. Bacteriophages were isolated as previously described with slightly modification (Megha et al., 2017). Briefly, 8 g of each sample were mixed with 1 mL of LB broth and 1 mL of a culture of indicator *E. coli* strain (CNCTC 6896 or CNCTC 6246) in exponential phase (OD_600_ = 0.2–0.3). The suspension was incubated overnight at 37°C with shaking (120 rpm) and then centrifuged at 13,000 *g* for 10 min at 4°C (Rotina 380 R, Hettich, Tuttlingen, Germany). The supernatant was filtered through a 0.45 μm pore size cellulose acetate syringe filter (Sartorius, Göttingen, Germany).

The crude filtrate was analyzed for the presence of phages *via* spot-test. Five mL of LB soft agar (0.5%) supplemented with CaCl_2_ 0.01 M were mixed with 100 μL of exponential-phase culture *E. coli* strains CNCTC 6896 or CNCTC 6246 and poured on LB bottom agar (1.5%) to create a double layer. Then, 10 μL of each filtrate were spotted onto the agar surface and plates were incubated overnight at 37°C. A clear zone of bacterial lysis denoted the presence of phages.

The supernatants containing phages were then decimally diluted in LB broth, 100 μL of which were mixed with 100 μL of exponential-phase of *E. coli* indicator culture and incubated at 37°C for 15 min. After incubation, mixtures were suspended in 5 mL of melted LB soft agar (0.5% w/w agar) supplemented with 0.01 M CaCl_2_ and poured onto LB bottom agar (1.5% w/w agar). Plates were then incubated overnight at 37°C. Well-separated plaques were picked up with a sterile Pasteur-pipette, transferred to a tube containing100 μL of exponential-phase of indicator *E. coli* culture along with 10 mL of LB broth supplemented with CaCl_2_ and incubated overnight at 37°C. Afterward, samples were centrifuged at 6,700 *g* for 10 min and filtered through a 0.45 μm pore size cellulose acetate syringe filter (Sartorius). The filtered suspensions were stored at 4°C. The phage titer of each viral suspension was assessed as stated above and the number of plaque forming units (PFU/mL) was calculated.

### Host Range Analysis

The host range of each phage was determined through a spot assay as described above, using exponential-phase STEC strains listed in Table 1. Briefly, 10 μL aliquot of each phage suspension were spotted onto each bacterial overlay and incubated overnight at 37°C. Plaque formation was evaluated according to lysis intensity. The experiment was performed in triplicate. Results were used to formulate a mixed viral suspension containing three different bacteriophages, named “cocktail.” The concentration of viral particles in the mixture was the same for each phage (about 10^7^ PFU/mL) and it was prepared in order to obtain the expected MOI.

### Bacterial Cell Lysis Assay

The lytic effect of the phage cocktail on STEC strains was assessed through the measurement of optical density (OD) at 600 nm (7315 Spectrophotometer, Jenway, Stone, United Kingdom). Phage cocktails were added to LB broth supplemented with CaCl_2_ containing exponential-phase STEC strains (ca 7.5 × 10^8^ cells/mL) to different Multiplicity of Infection (MOI): 0.1, 1, and 10. The suspensions were incubated at 37°C and OD_600 *nm*_ was measured at 0 and every 60 min over 6 h. A positive control sample was carried out by inoculating each bacterial strain without adding any phage cocktail. A negative control sample consisting of inoculated phage cocktail without any bacteria was also included in each assay. In order to normalize and compare the results obtained in various experiments, the value of the “area under the curve” (auc) of optical density formed by the growing of bacteria in 6 h was determined. This value integrated the carrying capacity, the growth rate and the contribution of initial population in a single data (Sprouffske and Wagner, 2016).

The data were analyzed using R Core Team (R Core Team, 2017) software packages “Growthcurver” and “ggplot2” for graphic elaboration. The ANOVA of the data was elaborated with Statgraphics Centurion (v. 18, Statistical Graphics Corp., Herndon, VA, United States); the Tukey’s HSD test wad used to compare the sample means in order to evaluate significant differences.

### Bacteriophage DNA Extraction

To obtain high-titer phage stock solutions, 50 mL of lysate were precipitated by adding 10% (w/v) of polyethylene glycol 6000 (PEG) (Merck, Darmstadt, Germany) in 0.5 M NaCl (final concentration) at 4°C for at least 4 h. Thereafter, samples were centrifuged at 8,000 *g* for 20 min, to allow phage precipitation. The precipitate was resuspended in 400 μL of Sodium-Magnesium (SM) buffer (0.05 M Tris-HCl buffer, pH 7.5, 0.1 M NaCl, 0.008 M MgSO4, and 0.01% gelatin) by shaking at 120 rpm for 4 h at 25°C. Then, 20 μL of EDTA solution (0.5 M; pH 8.0), 50 μL of SDS 10% (w/v) and 5 μL of proteinase K (Sigma-Aldrich, St. Louis, United States, final concentration 50 μg/mL) were added and phage lysates were incubated at 37°C for 1 h. Finally, standard phenol-chloroform DNA purification with ethanol precipitation was carried out to obtain purified phage DNA (Sambrook and Russell, 2001). Samples were stored at −20°C until use.

### Assessment of the Presence of Toxin Genes and RAPD Fingerprinting

For the new isolated phages the presence of genes encoding Shiga-like toxins (*stx1, stx 2, stx2f*) and intimin (*eae*) was assessed by polymerase chain reaction (PCR), according to EU-RL VTEC_Method_01_Rev 0 (2013) protocol. DNA from a temperate phage of O26 STEC strain F1-1 was used as a positive control. Multiplex-PCR reactions were set up in a 25 μl final volume containing: 10x buffer with MgCl2, dNTPs 0.2 mM, 25 pmol of each primer (Paton and Paton, 1998; Scheutz et al., 2012) 1 U of Taq polymerase (Biotechrabbit, Hennigsdorf, Germany) and 1 μl of template DNA. The thermal profile consisted in 35 PCR cycles (1 min of denaturation at 95°C; 2 min of annealing at 65°C for the first 10 cycles, decrementing to 60°C by cycle 15; 1.5 min of elongation at 72°C, incrementing to 2.5 min from cycle 25–35). Random amplification of polymorphic DNA (RAPD) was carried out with M13 primer (5′-GAGGGTGGCGGTTCT-3′) (Huey and Hall, 1989) at a final concentration of 0.5 mM with the same PCR reaction mix reported before. Thermal parameters for denaturing, annealing, and extension temperatures were 94°C for 2 min, 94°C for 20 s 35°C for 20 s for 40 cycles and a final elongation at 72°C for 2 min. The PCR products were subjected to electrophoresis in 1% agarose gel in 1X TAE buffer (Tris-acetate 40 mM, ETDA 1 mM, pH 8) added with 0.4 μg/mL of ethidium bromide with a 100 bp DNA ladder (BiotechRabbit, Henningsdorf, Germany) at a voltage of 80 V for 1.5 h prior to visualization with UV transilluminator (GELDOC XR-System, Bio-Rad Laboratories, Hercules, United States).

### Efficacy of the Phage Cocktail Against STEC in Fresh Cucumber

In order to evaluate the capability of the phage cocktail to reduce STEC contamination in fresh produce, a challenge test was set up slightly modifying the protocol of Snyder et al. (2016). Fresh cucumbers were purchased from a local market and thoroughly washed to remove any soil trace. Cucumbers were sliced and cut in pieces of ~3 g and both sides were treated with a UV lamp for 1 h to reduce the background microbiota. The pieces were then divided in three batches of about 10 g each and placed in Petri dishes. Two were spotted with 10 μl of a pathogenic *E. coli* culture in exponential phase (0.2 OD) at a concentration of 1 × 10^6^ CFU/mL. Then, one batch was dipped for 2 min in a beaker containing 50 mL of the three phages (FM10, DP16 and DP19) at the same titer (1 × 10^7^ PFU/mL). The other batch was dipped in the same solution without phages. All the pieces were allowed to dry for 1 h in a biosafety cabinet, transferred in sterile plastic box, and stored at 4 and 25°C. Bacterial counts were carried out at the beginning (t_0_), after 6 (t_6_), and 24 h (t_24_). The cucumber pieces were diluted in Tryptone Salt (1 g L^–1^ Tryptone, 9 g L^–1^NaCl) and homogenized in a Stomacher (Colsworth 200) for 2 min. Appropriate dilutions of the samples were then plated in TBX agar plates (Scharlau, Sentmenat, Spain). Three different experiments were run. The non-contaminated batch was analyzed to evaluate the bacterial count after UV treatment using the same protocol but measured only at t_0_.

## Results

### Antibiotic Resistance Assay

Six antibiotics were tested, namely amoxicillin/clavulanic acid, ciprofloxacin, norfloxacin, chloramphenicol, ampicillin, and nalidixic acid. Each of the thirty-one STEC strains, submitted to disk diffusion susceptibility test, showed sensitivity to at least one of the antimicrobial compounds investigated. Data were interpreted according to parameters proposed by EUCAST (EUCAST, 2019). Strains 228GS (O145), ED238 (O121), and PO128 (O128) showed the widest resistance, being inhibited by only one antibiotic out of six (MAR = 0.83). On the other hand, 6182-50 (O113) and F95-3 (O26) were sensitive to all six compounds (MAR = 0.00) (Table 1). Ampicillin, an antibiotic used in human medicine for the treatment of coliform infections, was the most effective antimicrobial agent, showing inhibition on 20 STEC strains out of 31 (65%), while nalidixic acid showed the lowest efficacy by inhibiting only 10 strains out of 31 (32%) (Table 1). The disk containing a solution of 50% (v/v) ethanol did not produce any inhibition, showing that the observed efficacy of the chloramphenicol solution was not due to the presence of the alcohol. No correlation among serogroup and antibiotic resistance was demonstrated.

### Isolation of Bacteriophage and Host Range Determination

A total of 20 *E. coli* bacteriophages were collected; in particular, 15 phages were isolated from 15 bovine feces samples, 2 phages from 2 bovine bedding material samples and 3 phages from 3 sewage samples. FM3, FM6, FM8, FM10, DP13, DP14, DP15, DP16, DP17, DP18, DP19, and DP20 phages were detected and purified by using the indicator strain *E. coli* CNCTC 6896 whereas FM1, LF2, LF4, FM5, LF7, FM9, FM11, and LF12 phages with the indicator strain CNCTC 6246, respectively. No active viral particles could be recovered from 2 samples coming from ovine matrices. During the isolation process, plaques with different morphology were collected from plates at highest dilutions assuming that phages present at elevated titers would be more likely to display a lytic biological lifestyle.

**FIGURE 1:**
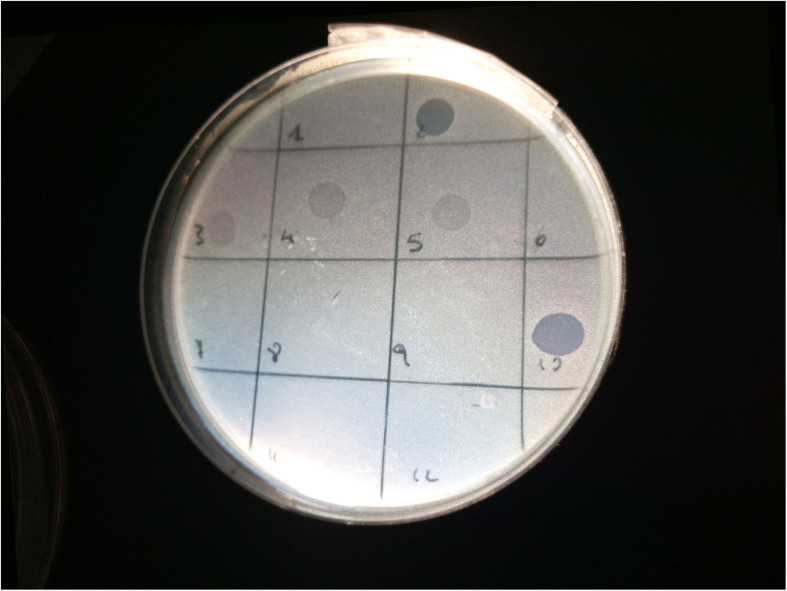
Image of spot test for the bacterial strain 33C (serogroup O23): phages LF2 (square 2) and FM10 (square 10) produced clear lysis plaques; phages FM3 (square 3), LF4 (square 4), and FM5 (square 5) generated turbid plaques; phages FM1 (square 1), FM6 (square 6), LF7 (square 7), FM8 (square 8), FM9 (square 9), FM11 (square 11), and LF12 (square 12) did not show any lysis.

**FIGURE 2:**
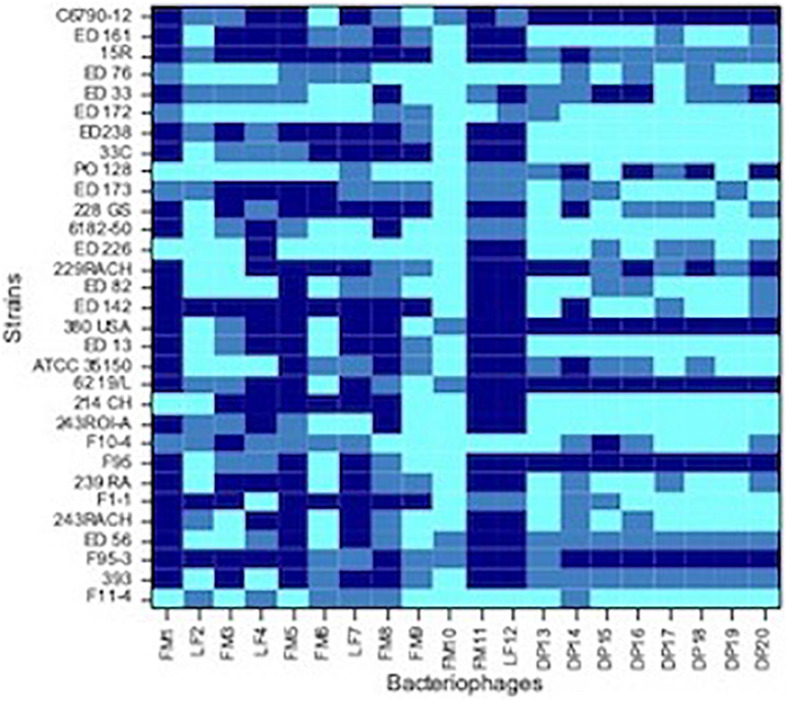
Heat-map showing the host range of each isolated phages. Dark blue, no sensitive strain; medium blue, strain with low sensitivity; light blue, strain with high sensitivity.

Spot tests were performed to assess the ability of the isolated viral particles to infect and lyse thirty-one STEC strains previously collected from different sources. Strain sensitivity to each phage was evaluated by observing the type of clarification zone onto double layer LB agar plates: the formation of clear plaques was interpreted as high sensitivity to the phage, while that of turbid plaques as low sensitivity (Figure 1). FM10, isolated from bovine feces, was the bacteriophage with the broadest host range, being able to infect all the 31 STEC strains. Eleven phages (55%) showed to be active on more than 70% of examined strains. Among these, the most promising ones were LF2, FM9, DP13, DP15, and DP20 (Figure 2). Therefore, a viral suspension containing a controlled mixture of these bacteriophages with different host ranges could potentially be effective at inhibiting all the tested STEC strains. On the other side, phages FM1, FM5, FM11, and LF12 exhibited narrow spectra of activity, infecting 8 to 10 strains and suggesting that they could not be the optimal choice for a cocktail formulation.

As concern strains, *E. coli* F95-3 (O26) and 380USA (O157) showed to be most resistant ones being sensitive to only 5 phages out of 20.

### Assessment of Bacterial Inactivation Kinetics

Basing on the data obtained from the host range assay, a cocktail containing phages FM10, DP16 and DP19 at the same titer (PFU/mL), was formulated. The effect of three different MOI’s was investigated (0.1, 1, and 10) by monitoring the optical density of mixed suspensions through hourly measurements.

**FIGURE 3:**
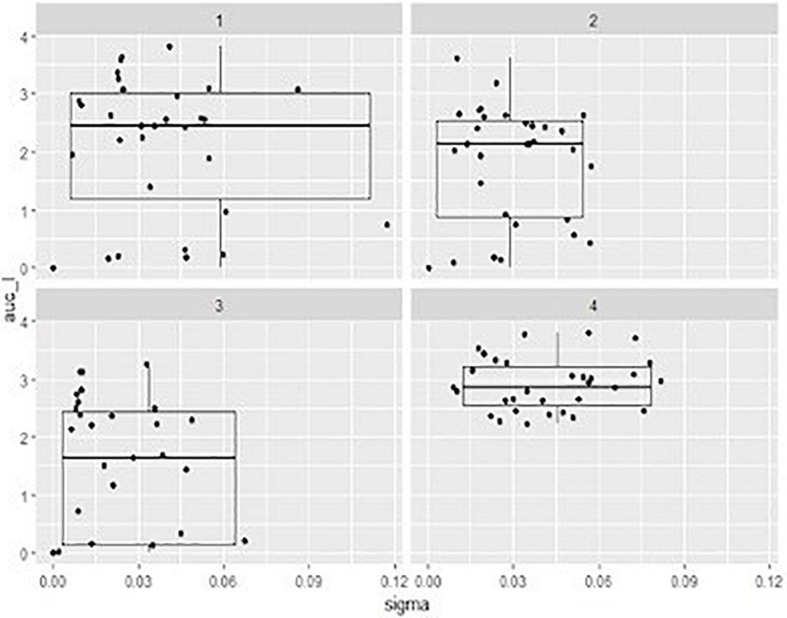
Box-plots representing the distribution describing the of area under the curve (auc) values ont x axe and the sigma value (the residual sum of squares from the non-linear regression model) onto y axe showed by mixed suspensions of the phage cocktail with each STEC strain, at three different multiplicity of infection (MOI) and relevant sigma value. 1, MOI 0.1; 2, MOI 1 3, MOI 10; 4, positive control.

In order to compare the activity of the phage cocktail on each STEC strain throughout the incubation period, the value of the area under the curve (auc) was calculated by fitting the experimental OD points with software packages cited in “Materials and Methods” section. It can be observed that, in general, a higher MOI corresponds to a more effective cell growth reduction (Table 2), represented by a lower value of auc showed by the development of bacteria over 6 h (Figure 3).

**TABLE 2:** Values of area under the curve (auc) obtained by adding the phage cocktail to each strain with different Multiplicity of Infection (MOI).

Strain	MOI 0.1	MOI 1	MOI 10	Control
ATCC35150	0.18	0.14	0.13	3.05
393	2.61	2.34	0.76	2.69
15R	1.40	0.84	0.00	2.35
214CH	2.59	2.18	1.17	2.64
221RACH	3.09	2.45	2.20	3.00
228GS	0.17	2.02	0.00	3.04
239RA	3.81	2.49	2.37	3.78
243RACH	2.43	2.64	1.65	2.42
243RoI-A	3.08	2.04	1.51	2.97
33C	0.97	0.56	0.73	3.27
380USA	2.80	2.71	2.80	2.79
6182-50	3.58	2.35	0.00	3.80
62 19/L	3.62	2.41	3.13	3.53
C679-12	0.32	0.00	0.00	2.35
ED13	0.22	0.18	0.00	2.65
ED142	2.45	2.14	1.44	2.44
ED161	0.75	2.14	0.21	2.26
ED172	2.20	1.94	2.50	3.28
ED173	3.25	3.18	3.25	3.14
ED226	3.37	2.74	2.29	3.33
ED238	2.87	2.60	2.74	2.86
ED33	1.88	1.76	0.16	2.45
ED56	3.08	2.64	3.12	3.45
ED76	1.94	1.46	2.39	3.70
ED82	2.95	2.14	0.00	2.95
F1-1	0.21	0.10	1.69	2.23
F10-4	2.24	0.92	2.13	2.63
F11-4	2.56	0.74	0.00	3.08
F95	2.45	2.43	2.49	2.39
F95-3	2.57	2.62	2.22	2.63
PO128	0.00	0.43	0.34	2.85
Mean	2.12^*b*^	1.78^*a,b*^	1.40^*a*^	2.91^*c*^

*Values with different superscript letters (a–c) are significantly different (P < 0.05).*

The auc mean value of positive controls (2.91) was significantly higher (*p* < 0.05) than the auc mean value of the experiments with the addition of phage cocktail; finally, with MOI at 0.1 the auc mean value was 2.12, with MOI at 1 was 1.78, and with MOI at 10 was 1.40. Moreover, the auc mean value of the experiments with MOI at 0.1 was different (*p* < 0.05) from that one obtained with MOI at 10.

Also, the distribution of the auc values varied considering the different MOI used. In particular, while results of positive controls are clustered in a limited area, data of the other groups were widely distributed due to the fact that some strains are resistant to the phage cocktail and, in these cases, the auc values are similar to values obtained from controls (Table 2).

The value of sigma (i.e., the residual sum of squares from the non-linear regression model) revealed the goodness of the fit with parameters of the logistic equation for the observed data. In our case sigma value ≤ 0.12 can be considered as a good fitting. Considering indeed that the expected cell growth of strains was that described by the positive controls, the addition of phage cocktail significantly changed the behavior of the suspensions with a lower value of sigma close to 0 for the highest MOI (Figure 3).

Nevertheless, six out of 31 STEC strains proved to be resistant to the phage cocktail (Table 2). In some cases, such as for strains ED82, ED226, 214CH, ED142, ED33, and 243RACH, a MOI of 10 was necessary to observe substantial reduction of bacterial cell concentration. Otherwise, including some of those in first category, the application of a high MOI reduced the lytic effect of the cocktail.

### Assessment of the Presence of Toxin Genes and RAPD Analysis

Since bacteriophages play a major role in horizontal gene transmission, the DNA extracted from each phage was examined by PCR for the presence of specific STEC virulence factors such as genes encoding Shiga toxins and intimin. No amplification was obtained from any sample, while the positive control exhibited amplification signals at the expected sizes, demonstrating that none of the twenty phages carried these genes. A RAPD analysis was performed to highlight potential similarity among phage isolates. In order to recognize if viral DNAs were contaminated with bacterial DNA of the host, amplifications were carried out also on DNA extracted from the two indicator strains. In Supplementary Figure S1 results from the amplification on the three selected phages are reported showing different and reproducible fingerprints. Viral DNA samples revealed different patterns in comparison to their respective propagation bacteria, so the observed differences are likely attributed to dissimilarity among phages. Moreover, the DNAs from the three selected phages were subjected to a complete genome sequencing (personal communication of prof. David Pride, University of San Diego, United States) that confirmed the absence of virulence-encoding genes and antibiotic-resistance genes.

### Preliminary Testing on Inoculated Fresh Produce

A first trial to evaluate the efficacy of the phage cocktail to control STEC in fresh produce was done using cucumber as model system. Samples after washing and UV treatment did not show a residual bacterial count (< 10 CFU/g). Fresh cucumber slices were artificially contaminated with ~10^3^ CFU/g of ATCC35150 (O157:H7) sensitive strain. Treatment with the phage cocktail lead to a reduction of bacterial counts of 1.97 and 2.01 log CFU/g at 25°C and of 1.16 and 2.01 log CFU/g at 4°C, after 6 and 24 h, respectively (Table 3).

**TABLE 3:** Average of ATCC35150 microbial count of three replicates at three time, at two different temperature express in log CFU/g.

	25°C		4°C	
	
ATCC35150	Control	Cocktail	Control	Cocktail
t0	2.88 (± 0.24)		2.88 (± 0.24)	
t6	4.06 (± 0.84)	2.09 (± 0.45)	3.11 (± 0.17)	1.95 (± 0.41)
t24	9.01 (± 0.04)	7.00 (± 0.44)	3.26 (± 0.52)	1.25 (± 1.09)

**Standard deviation is showed in parentheses.**

## Discussion

Twenty phages active on STEC strains were isolated from cattle feces, bedding material and sewage, indirectly confirming that bovine gut is a natural reservoir for these pathogens and often the main route of contamination for raw materials and dairy products, especially when prepared in inadequate hygienic conditions. The relative high number of virions isolated in this study from a small collection of samples corroborates these substrates as a consistent source of *E. coli* bacteriophages. A preliminary characterization pointed out viral populations showing different plaque morphologies and host ranges. The formation of turbid plaques in few strains could be due to the presence of resistance phenomena such as abortive infection mechanisms (Dy et al., 2014) which prevent the spread of progeny virions and thus protect clone cells from infection.

Although phage FM10 proved to be able to lyse all the STEC strains used in these study, we decided to use a cocktail of different bacteriophages to be more effective and reduce the emergence of phage resistance (Goodridge and Abedon, 2003). The choice of using three phages seemed, in accordance with literature (O’Flynn et al., 2004; Bai et al., 2019; Yin et al., 2019) a good compromise considering the possibility of phage recombination and the generation of new host specificity, and even the high production costs. Besides, according to the “Red Queen hypothesis” (“It takes all the running you can do, to keep in the same place”), as bacteria develop phage defense mechanisms for their survival, phages continuously adapt to these altered host systems in order to avoid a complete destruction (Lythgoe and Read, 1998).

The lytic ability of our phage cocktail has been assessed at different MOIs. As expected, in 61% of cases the higher MOI allowed an effective reduction of the bacterial population. A lower performance was observed in experiments with few strains (380USA, 6219/L, ED56, and F95-3), probably because no phage, present in the mixed suspension, had been capable to form clear lysis plaques on them in the previous screening tests. The behavior of the phage cocktail on ED173 and F95 strains is difficult to interpret since they did not show any lowering of the auc values although they revealed sensitivity to the attack by phage F10 when alone. STEC strains are mostly lysogenic and, therefore, they continuously synthesize repressor proteins to maintain its lifestyle which can inhibit further infection. Moreover, the current MOI drives the decision made by the phage when its DNA is injected into the host cell (Blotnick et al., 2018) in this work we have not evaluated which intracellular events may have occurred. However, it has been confirmed that the choice of bacteriophages forming clear lysis plaques, probably going in virulent cycle, is preferable to those that generate turbid plaques. The phage cocktail used in this work achieved a 2 log reduction of *E.coli* cells after 24 h incubation both at 4 and 25°C making it a promising tool for the biocontrol of STEC on fresh produce.

No correlation was observed between host range and serogroup or antibiotic resistance spectrum. Antibiotics are not allowed for food applications but the presence of antibiotic resistant pathogens on these substrates are considered a risk for public health. However, control of antibiotic resistant pathogens is a global challenge, especially considering the difficulty in developing new classes of antimicrobials. The high resistance evidenced in our strains to nalidixic acid (a quinolone antibacterial agent for oral administration) and ciprofloxacin (a second-generation fluoroquinolone), both used to treat *E. coli* infections, reinforces the hypothesis that the use of these molecules against STEC strains might be ineffective. Antibiotic and phage resistance are provided by different mechanisms suggesting that the formulation of a phage cocktail active on different STEC strains that can be used on crops can help in prevent foodborne disease and the subsequent treatment of patients with inefficient antibiotics. Furthermore, a number of bacteriophage cocktails have been already granted as Generally Recognized as Safe (GRAS) by the FDA and already available on the market (e.g., SalmoFresh^TM^, ListShield^TM^, and PhagheGuard S^TM^) (Moye et al., 2018). Our phage formulation showed to inhibit strains 228G and PO128 that exhibited resistance to different antibiotics and to reduce significantly all other tested STEC strains.

## Data Availability Statement

The raw data supporting the conclusions of this article will be made available by the authors, without undue reservation.

## Author Contributions

NM and CP planned the experiments and wrote the manuscript. NM and RC performed the experiments and analyses. NM and RF processed the data. RF and CP revised the manuscript. All authors contributed to the article and approved the submitted version.

## Conflict of Interest

The authors declare that the research was conducted in the absence of any commercial or financial relationships that could be construed as a potential conflict of interest.
