# Supplementary figures and images for: Evaluation of a Potential Bacteriophage Cocktail for the Control of Shiga-Toxin Producing Escherichia coli in Food
Source: Front Microbiol. 2020 Jul 24;11:1801. doi: 10.3389/fmicb.2020.01801 (PMC7393728; doi:10.3389/fmicb.2020.01801)

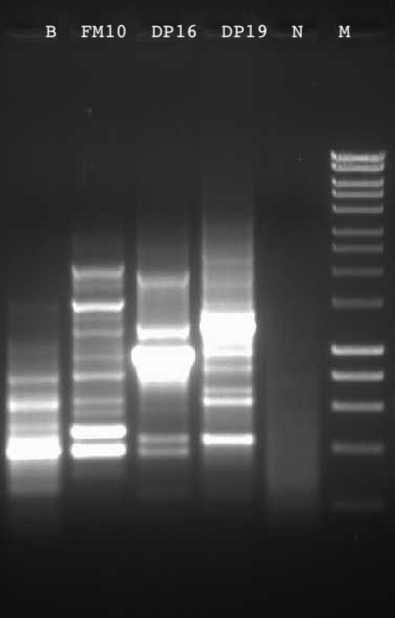

Supplement: Supplementary file 1 [file Image_1.JPEG]
