# Supplementary material for: Evaluation of a Potential Bacteriophage Cocktail for the Control of Shiga-Toxin Producing Escherichia coli in Food
Source: Front Microbiol. 2020 Jul 24;11:1801. doi: 10.3389/fmicb.2020.01801 (PMC7393728; doi:10.3389/fmicb.2020.01801)
Supplement: Supplementary file 2 [file Table_1.DOCX]

**Fig S1** RAPD analysis on bacteriophages; B: bacterium in which the bacteriophages are propagated (CNCTC 6896); N: sample without DNA; M: all sizes marker (LeGene Biosciences, San Diego, USA).
